# Efficacy and mechanisms of immune checkpoint inhibitors in late-stage EGFR-mutated non-small cell lung cancer following targeted therapy resistance

**DOI:** 10.3389/fimmu.2026.1845547

**Published:** 2026-06-03

**Authors:** Jiling Niu, Wang Jing, Zongkai Liu, Yunxuan Si, Zhaidong Liu

**Affiliations:** 1 Department of First Clinical Medical College, Shandong University of Traditional Chinese Medicine, Jinan, China; 2 Department of Oncology, Affiliated Hospital of Shandong University of Traditional Chinese Medicine, Jinan, China

**Keywords:** EGFR mutations, immune microenvironment, immunotherapy, non-small cell lung cancer, resistance, tyrosine kinase inhibitors

## Abstract

Currently, epidermal growth factor receptor-tyrosine kinase inhibitors (EGFR-TKIs) represent the standard first-line treatment for advanced EGFR-mutated non-small cell lung cancer (NSCLC). However, as the disease progresses, targeted resistance inevitably develops. Chemoimmunotherapy, as the standard treatment for advanced NSCLC without driver gene mutations, has significantly improved patient survival. EGFR-mutant tumors exhibit unique immunogenicity compared to wild-type tumors, with heterogeneity in programmed death ligand 1(PD-L1) expression levels, tumor mutational burden (TMB), and other immune microenvironment characteristics. Therefore, we elucidate the mechanisms of immune resistance in EGFR-mutant patients and analyze the core immune mechanisms underlying EGFR-TKI resistance. We summarize the application of immune checkpoint inhibitors (ICIs) in advanced EGFR-mutant NSCLC and analyze the associated mechanisms of action.

## 1 Introduction

Lung cancer, as the cancer type with the highest incidence and mortality rates globally, severely impacts human health and survival (1). Non-small cell lung cancer (NSCLC) accounts for approximately 80-85% of all lung cancers (2). Epidermal growth factor receptor (EGFR) mutations represent the most common mutation type in NSCLC, occurring in about 30% of cases (3, 4). In adenocarcinomas, this proportion can reach as high as 40-60% (4–6).

Treatment for EGFR-mutant advanced NSCLC has entered the era of targeted therapy, achieving a three-stage leap in progression-free survival (PFS). The median progression-free survival (mPFS) for first-generation epidermal growth factor receptor-tyrosine kinase inhibitors (EGFR-TKIs) ranges from 9.2 to 13.1 months (7–11). Second-generation EGFR-TKIs demonstrated a mPFS of 11 to 14.7 months (12, 13). Third-generation EGFR-TKIs achieved a mPFS of 17.8 to 22.1 months (14–17). Furthermore, the novel third-generation EGFR-TKI (Limertinib) demonstrated a median second-line progression-free survival (mPFS2) of 11.0 months in patients with T790M mutation progression after EGFR-TKI therapy, also exhibiting favorable efficacy (18). Consequently, third-generation EGFR-TKIs have become the standard first-line treatment for advanced EGFR-mutated NSCLC (15, 17, 19).

Targeted therapy has significantly improved efficacy, but resistance to EGFR-TKIs inevitably develops, typically occurring within 10–22 months (16, 20–22). Treatment options after targeted resistance vary, with differing degrees of benefit. After EGFR-TKI resistance, platinum-based dual-agent chemotherapy is the primary approach, offering limited clinical benefit with a PFS of only 4–5 months (23–25). The IMPRESS study demonstrated that extending TKI therapy beyond resistance development, compared to chemotherapy, did not improve overall survival (OS) or PFS (24, 26). Current efficacy of anti-angiogenic agents combined with chemotherapy: mPFS ranges from 2.8 to 6.6 months, mOS of 14.2 to 18.2 months (27, 28). Treatment regimens including chemotherapy alone, chemotherapy plus targeted therapy, or chemotherapy plus anti-angiogenic agents have not significantly improved outcomes for EGFR-mutant patients after targeted therapy resistance.

Today, with the successive introduction of immune checkpoint inhibitors (ICIs), anti-programmed cell death protein 1/programmed death-ligand 1 (PD-1/PD-L1) antibodies have been approved for treating patients with advanced NSCLC. Compared to traditional chemotherapy, ICIs offer greater clinical benefits for patients without sensitive gene mutations (29–32). While combination therapy of chemoimmunotherapy has been well-documented in patients with advanced EGFR-TKI-resistant disease, the role of ICIs in this population remains unclear.

This paper elucidates the mechanisms underlying immune resistance in patients with EGFR mutations and analyzes the core immune mechanisms driving resistance to EGFR-TKIs. It reviews clinical trials of ICIs in patients with advanced EGFR-mutant NSCLC, explores potential beneficiary populations, and examines the advantages and disadvantages of different treatment modalities. Furthermore, it envisions future research directions for immunotherapy in patients with EGFR-TKI-resistant disease.

## 2 Mechanisms linking EGFR mutations to ICIs resistance

**Figure 1:**
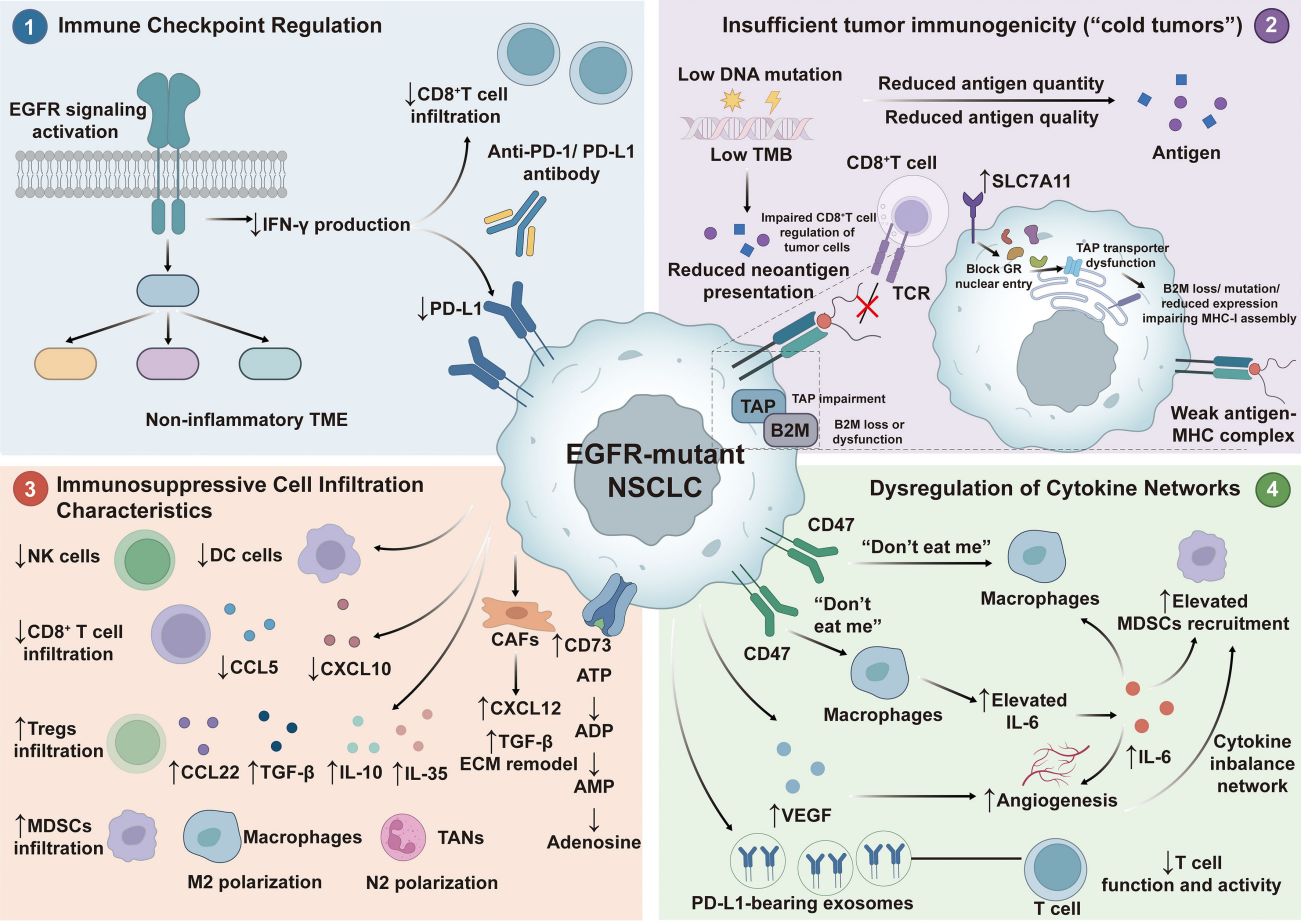
Mechanisms linking EGFR mutations to ICIs resistance.

EGFR-mutated NSCLC generally exhibits poor response to ICIs, with resistance mechanisms involving multidimensional remodeling of the tumor microenvironment (TME) (**Figure 1**).

### 2.1 Immune checkpoint regulation

PD-L1 expression in EGFR-mutated tumors exhibits high heterogeneity and lacks consistent correlation with ICI efficacy (33, 34). Their microenvironment is characterized as a “non-inflammatory TME,” defined by deficient CD8+ T cell infiltration (35). Studies indicate that the EGFR signaling pathway directly suppresses cytokine secretion, such as interferon-γ (IFN-γ), thereby downregulating PD-L1 expression and weakening the regulatory function of immune checkpoint molecules (35, 36). This immunosuppressive microenvironment leads to generally limited clinical response rates with anti-PD-1/PD-L1 antibody monotherapy (36).

### 2.2 Insufficient tumor immunogenicity (“cold tumors”)

EGFR-mutant NSCLC typically exhibits low tumor mutational burden (TMB), resulting in insufficient neoantigen production that impairs the immune system’s recognition and attack capabilities (33, 34). Immunogenicity depends not only on the number of mutations, but even more so on the number of high-quality immunogenic neoantigens. The neoantigens generated by EGFR-mutated tumors are difficult for T cells to recognize due to their low affinity and poor presentation (37). In addition, during early tumor development, highly immunogenic clones may have been eliminated through immune selection, leaving behind residual clones with new antigens of generally lower “quality.” (38). Defects in antigen presentation and processing are major contributors to “cold tumors.” Overexpression of EGFR can stably regulate SLC7A11 expression through a non-kinase-dependent mechanism, thereby inhibiting the antigen-presenting function of MHC-I molecules and impeding CD8^+^ T cell recognition of tumor cells (39). The loss of MHC-I expression is a key mechanism of tumor immune evasion (40). B2M is an essential light chain of the MHC-I complex; its gene deletion, mutation, or reduced protein expression can lead to failure of MHC-I assembly, allowing tumor cells to evade surveillance by CD8^+^ T cells (41–43). The transporter associated with antigen processing (TAP) is responsible for transporting antigenic peptides from the cytoplasm to the endoplasmic reticulum for loading onto MHC-I (44). TAP dysfunction can lead to the failure of the classical antigen-presentation pathway, resulting in ICI resistance (44). EGFR signaling plays a role in the stable expression of SLC7A11. The high-level, stable expression of SLC7A11 blocks the entry of the glucocorticoid receptor (GR) into the cell nucleus, thereby inhibiting the transcription of TAP1 and the presentation of MHC-I molecules (39).

### 2.3 Immunosuppressive cell infiltration characteristics

EGFR-mutated NSCLC exhibits an immunosuppressive microenvironment characterized by reduced CD8+ T cell infiltration and increased regulatory T cells (Tregs) infiltration (45). EGFR signaling downregulates the chemokines CCL5 and CXCL10 in CD8+ T cells while upregulating CCL22 in Tregs (45). Furthermore, EGFR-mutated tumor cells may upregulate CD73 to convert ATP into adenosine (ADO), thereby activating the ADO pathway to enhance Treg expression. This alters the function of both tumor cells and immune cells, generating an immunosuppressive TME (46). Treg-secreted TGF-β, IL-10, and IL-35 creates an immunosuppressive environment that weakens the antitumor effects of CD4+ T cells, CD8+ T cells, and NK cells (46). Amphiregulin (AREG), one of the EGFR ligands, binds to EGFR expressed on Tregs to activate EGFR signaling. This inhibits GSK-3β activity, promotes post-translational modification of Foxp3 protein, reduces Foxp3 degradation, and enhances Foxp3 expression, thereby sustaining the suppressive function of Tregs (46).

EGFR mutations disrupt the maturation process of dendritic cells (DCs), impairing their ability to activate CD8+ T cells and forming a key mechanism for immune escape (47). Single-cell transcriptomic analysis further reveals that, compared to EGFR wild-type tumors, EGFR-mutant tumors exhibit significantly increased infiltration of Tregs and myeloid-derived suppressor cells (MDSCs) (48). These immunosuppressive cells suppress effector T cell function by secreting factors such as TGF-β and IL-10, while simultaneously promoting M2-type tumor-associated macrophage polarization, collectively sustaining an immune-tolerant microenvironment (47, 48).

Furthermore, cancer-associated fibroblasts (CAFs) not only mediate resistance to EGFR-TKIs (49, 50), but also establish an immunosuppressive barrier through the CXCL12/TGF-β signaling axis and ECM remodeling (51–55). CAFs secrete the chemokine CXCL12, which inhibits CD8^+^ T-cell infiltration by enveloping tumor cells and “anchors” T cells to the stroma, preventing them from contacting tumor cells (51, 52). TGF-β is a key factor in CAF activation; its signaling pathway enhances immune exclusion: on the one hand, it promotes the secretion of chemokines such as CXCL12 by CAFs, and on the other hand, it suppresses cytotoxic T cell function, jointly establishing an immunosuppressive microenvironment (53, 54). As the central executors of ECM deposition and remodeling in the TME, CAFs form a physical barrier by constructing a dense fibrotic matrix, thereby impeding the infiltration of immune cells (55).

Tumor-associated neutrophils (TANs) are one of the primary infiltrating cell types in the NSCLC TME, and their abundance is closely associated with poor immunotherapy response and poor patient prognosis (56). TANs can exhibit either an antitumor N1 phenotype or polarize into a pro-tumor N2 phenotype, thereby playing a dual role in immune evasion, tumor metastasis, and the development of drug resistance (57). During tumor progression, TANs often polarize toward the N2 phenotype, migrating to the tumor core where they exhibit high expression of arginase, elastase, and various immunosuppressive factors, leading to impaired T-cell function and promoting the formation of an immunosuppressive TME (58).

### 2.4 Dysregulation of cytokine networks

Persistent activation of the EGFR signaling pathway leads to local dysregulation of tumor cytokine networks. Beyond the aforementioned suppression of IFN-γ secretion, EGFR mutations mediate a “don’t eat me” signal by upregulating CD47 expression, thereby aiding tumor cells in evading phagocytic clearance by the innate immune system (36). Furthermore, abnormally elevated factors such as VEGF and IL-6 in the TME promote angiogenesis and MDSC recruitment, further reinforcing the immunosuppressive state (36, 47). EGFR-mutant tumor cells can also secrete PD-L1-bearing exosomes that directly bind to T cells, thereby suppressing T cell function and activity (46). This multifactorial synergy constitutes the deep microenvironmental basis for the resistance of EGFR-mutant tumors to immunotherapy (36, 47).

## 3 Core immune mechanisms underlying EGFR-TKI resistance

### 3.1 Immune checkpoint regulation

**Figure 2:**
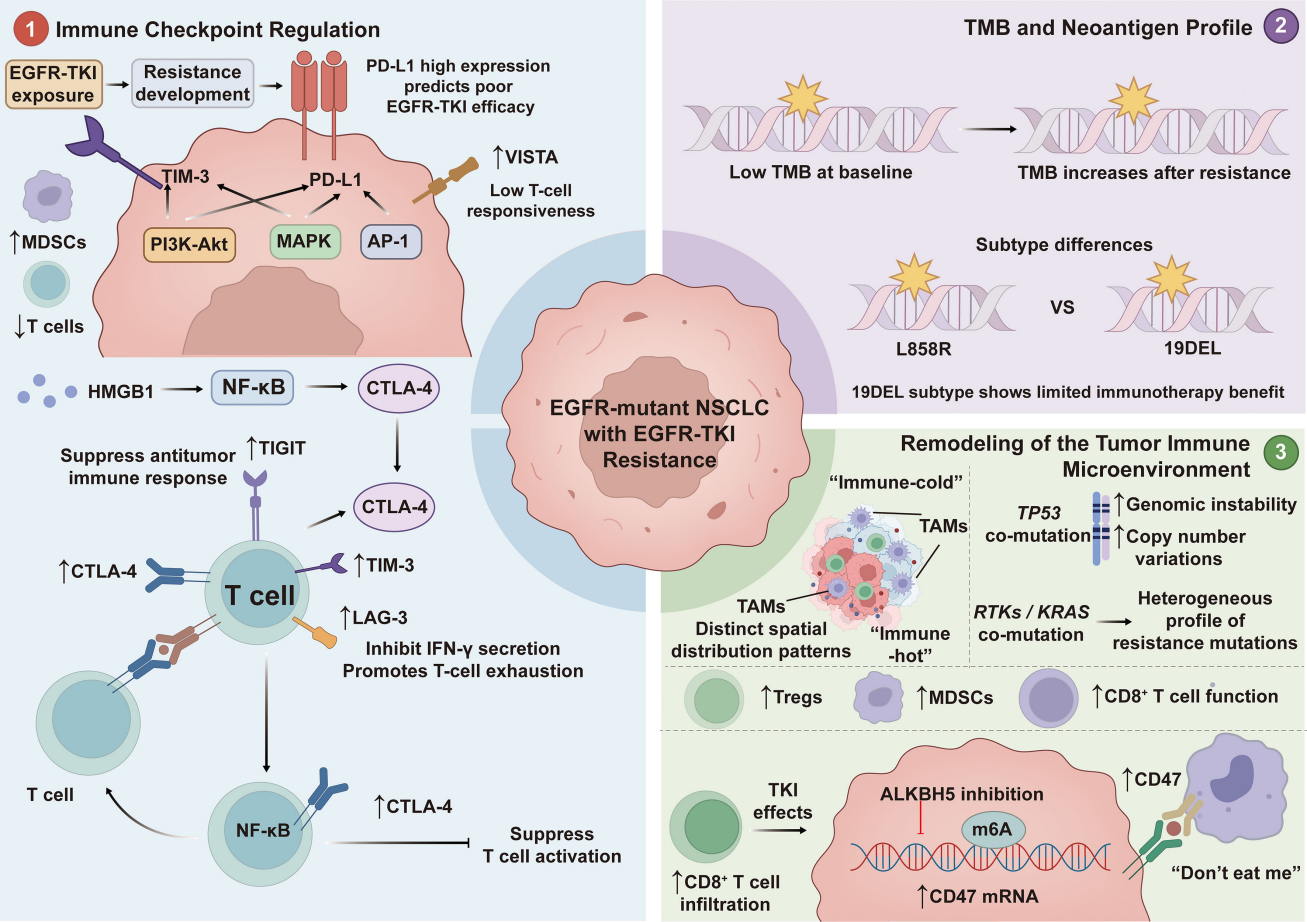
Core immune mechanisms underlying EGFR-TKI resistance.

In patients treated with first and second-generation EGFR-TKIs, PD-L1 expression can predict poor response to EGFR-TKIs and primary resistance. High PD-L1 expression is associated with more aggressive tumor biology and is closely linked to shorter PFS during EGFR-TKI therapy (59–61). In patients treated with third-generation EGFR-TKIs, high PD-L1 expression has also been shown to be associated with poor prognosis (62–65). Studies have shown that PD-L1 expression status differs before and after EGFR-TKI treatment, with PD-L1 expression levels increasing post-treatment (66–68). Acquired EGFR-TKI resistance promotes immune evasion in lung cancer by upregulating PD-L1 expression through the PI3K-Akt, MAPK, AP-1, and NF-κB signaling pathways (67) (**Figure 2**).

Furthermore, studies suggest that high cytotoxic T-lymphocyte-associated protein 4 (CTLA-4) expression may be a key driver of EGFR-TKI resistance (69). TKI therapy increases CTLA-4 expression on T cells in patients with brain metastases from EGFR-mutant lung cancer, thereby promoting the formation of an immunosuppressive microenvironment. Tumor-derived HMGB1 protein activates the NF-κB signaling cascade in T cells, leading to upregulation of CTLA-4 expression (69).

In addition, a new generation of immune checkpoint molecules is significantly upregulated and contributes to the establishment of an inhibitory immune microenvironment. lymphocyte activation gene 3 (LAG-3) is highly expressed in CD8^+^ T cells (including memory T cell subsets) and Tregs in TKI-resistant tumors (70). LAG-3 negatively regulates IFN-γ secretion and promotes T-cell exhaustion (71). Following TKI resistance, T-cell immunoglobulin and mucin domain-containing protein 3 (TIM-3) is upregulated via signaling pathways such as PI3K-Akt and MAPK, which are closely associated with increased infiltration of MDSCs and reduced T-cell infiltration within the tumor, constituting a key component of the immunosuppressive microenvironment (72). T-cell immunoreceptor with Ig and ITIM domains (TIGIT) is significantly overexpressed on the surface of NK cells and CD8^+^ T cells in resistant tumors, directly suppressing antitumor immune responses (73). V-domain Ig suppressor of T cell activation (VISTA) is specifically enriched in the immunosuppressive TME of EGFR-mutant NSCLC, with higher expression levels than in wild-type tumors, persistently maintaining a state of low T-cell responsiveness (74).

### 3.2 TMB and neoantigen profile

NSCLC with EGFR mutations typically has a low TMB, which increases significantly following TKI treatment (75). Similarly, in patients with EGFR-mutated colorectal cancer, TMB has been shown to increase significantly with TKI use (76, 77). Elevated TMB typically results from an increase in driver mutations and copy number variations; these alterations, occurring outside the EGFR pathway, may sustain tumor growth and undermine the efficacy of EGFR-targeted therapy. Higher TMB levels imply a greater number of potential neoantigens and stronger tumor immunogenicity, making such tumors more suitable for immunotherapy (78–80). Lung cancers with the EGFR L858R mutation exhibit higher TMB than those with the 19DEL mutation (75, 81). Immunotherapy benefits are limited in 19DEL-mutated lung cancers, whereas the L858R mutation demonstrates relatively better treatment efficacy (81). Multiple large-scale clinical studies have demonstrated that high TMB (typically defined as ≥175 mutations/exome or ≥10 mutations/Mb) is significantly associated with higher objective response rates (ORR), longer PFS, and longer OS following ICI treatment, and this association is independent of PD-L1 expression levels (82–85).

### 3.3 Remodeling of the tumor immune microenvironment

The immunosuppressive remodeling of the TME plays a key role in drug resistance. EGFR mutations can promote the formation of an immunosuppressive TME by interfering with intracellular signaling pathways and modulating immune helper cells (86), whereas treatment with EGFR-TKIs increases CD8+ T cell infiltration, reduces the number and activity of Tregs, and remodels the tumor immune microenvironment (TIME) (87). Studies have shown that EGFR-TKI resistance promotes the infiltration of immunosuppressive cells (such as regulatory T cells and myeloid-derived suppressor cells) and enhances immune checkpoint expression through epigenetic regulation (88, 89). The specific mechanism involves the inhibition of the RNA demethylase ALKBH5, leading to increased m6A modification levels of the CD47 molecule, which in turn activates the “don’t eat me” signal and suppresses the antitumor function of CD8+ T cells (89). This remodeling of the immune microenvironment provides a theoretical basis for combination immunotherapy.

Furthermore, following the development of resistance to EGFR-TKIs, the dynamic remodeling of the TME involves multidimensional mechanisms, including spatial heterogeneity, lineage plasticity, and co-mutation-driven immune regulation. The TME exhibits significant spatial heterogeneity, which directly influences the progression of resistance and treatment response. Tumor-associated macrophages (TAMs) exhibit distinct spatial distribution patterns within the TME and are involved in immune regulation, angiogenesis, tissue remodeling, and metastasis (90). This spatial heterogeneity leads to significant differences in the composition and functional states of immune cells across different regions within the same tumor, creating “immunologically cold” and “immunologically hot” zones, which in turn influence treatment outcomes (91). Lineage plasticity is one of the key non-genetic mechanisms underlying acquired resistance to EGFR-TKIs. For example, overexpression of FOXM1 combined with FOXA1 deletion can induce the transformation of lung adenocarcinoma into squamous cell carcinoma, reshaping the TKI-resistant phenotype (92). Epithelial-mesenchymal transition (EMT), as a typical manifestation of lineage plasticity, is also an important pathway for EGFR-independent resistance (93). Co-mutations influence the efficacy of EGFR-TKIs by reshaping the immune microenvironment. Patients harboring TP53 co-mutations and whole-genome duplication (WGD) exhibit increased genomic instability and higher copy number variations, which are associated with more complex resistance mechanisms and poorer prognosis (94). Exhibit co-mutations in receptor tyrosine kinases (RTKs) fusions, or driver genes such as KRAS, leading to a heterogeneous profile of resistance mutations that influences subsequent treatment selection (95).

## 4 Mechanisms of EGFR-TKI resistance are associated with the immune microenvironment

In the immune microenvironment of tumors resistant to EGFR-TKIs, the T790M mutation, MET amplification, and small cell lung cancer (SCLC) transformation are three common mechanisms of resistance. These mechanisms alter the tumor’s immunological characteristics through different pathways, thereby influencing immune evasion and the response to immunotherapy. T790M-positive tumors are typically associated with lower TMB and reduced immune cell infiltration, reflecting low immunogenicity (96). MET amplification, as an exogenous resistance mechanism to EGFR-TKIs, is often accompanied by higher TMB and more abundant immune cell infiltration. Activation of the MET signaling pathway promotes upregulation of PD-L1 expression in tumor cells, resulting in a more complex state of immune suppression. High TMB and PD-L1 expression increase the potential for an immune response, making MET-amplified tumors more sensitive to ICI combination strategies in certain cases (97). Transformed SCLC is often associated with enhanced immune suppression: compared to primary NSCLC, it exhibits lower T-cell infiltration, greater heterogeneity in PD-L1 expression, and active immune evasion mechanisms (98).

## 5 EGFR mutation subtypes are associated with the immune microenvironment

Overall, most EGFR mutation subtypes (19DEL, L858R, and Ex20ins) exhibit lower immunogenicity, characterized by lower PD-L1 and TMB expression, a higher proportion of immunosuppressive cells in the TME, and “cooler” TME immune cell profiles compared to wild-type tumors (99). Among classic mutations, the L858R subtype exhibits higher TMB, PD-L1 expression, and CD8+PD-1+ T-cell infiltration compared to the 19del subtype, suggesting that the L858R mutation subtype may be more sensitive to ICIs (100, 101). The distribution of immune cell infiltration, PD-L1, and TMB in Ex20ins tumors may be similar to that of classic mutations or lean more toward “cold tumors,” but there is currently no consensus (102). Tumor subtypes with rare mutations, such as those with L861Q and G719X mutations, exhibit higher TMB and TP53 co-mutation rates, as well as a lower proportion of immunosuppressive cells in the TME; consequently, their immunogenicity is relatively higher, making them more likely to benefit from ICI therapy (99).

## 6 Current status and future directions of immunotherapy following resistance to EGFR-TKIs

### 6.1 Monotherapy with immunotherapy

KEYNOTE-001: phase I/II clinical trials have shown that ICIs are significantly less effective in patients with advanced NSCLC harboring EGFR mutations than in patients with wild-type EGFR (103, 104) (**Table 1**). In the KEYNOTE-010 trial, when analyzing the PD-L1-positive population, the benefit observed in patients with EGFR mutations remained lower than that in wild-type patients (105). In the CheckMate-012 study, first-line treatment with Nivolumab showed no benefit, with a mPFS of only 1.8 months (106). Furthermore, in both the CheckMate-057 and OAK clinical trials, neither Nivolumab nor Atezolizumab demonstrated a survival benefit compared to chemotherapy (107, 108). Furthermore, two meta-analyses also demonstrated that monotherapy with ICIs offers no advantage in second-line treatment (109, 110). In patients with EGFR-TKI resistance, the mPFS in the Nivolumab monotherapy group was significantly lower than that in the platinum-based chemotherapy group, at only 1.7 months (111). In patients resistant to Osimertinib, the mPFS for immune monotherapy was only 1.5 months, and the mOS was 4.9 months (112). Results from the ATLANTIC study showed that mOS for monotherapy with an ICI was approximately 13.3 months in the subgroup of patients with EGFR mutations and TPS ≥ 25% following second-line chemotherapy (113).

**Table 1:** Efficacy of monotherapy immunotherapy in patients with EGFR mutations.

Study	PD-1/PD-L1 blockade therapy	Study type	Published year	Patients	Efficacy outcomes
Keynote-001 (NCT02879994) (103)	Pembrolizumab	Clinical Trial	2018	TKI Naïve, PD-L1-positive, EGFR-mutant NSCLC	Recruitment was halted due to a lack of efficacy.
Keynote-001 (NCT01295827) (104)	Pembrolizumab	Clinical Trial	2019	Locally advanced/metastatic NSCLC	Compared with patients with the wild-type EGFR, those with EGFR mutations had mOS of 6.0 months vs 11.9 months; the 5-year OS was 7.9 months vs 16.4 months.
Keynote-010 (NCT01905657) (105)	Pembrolizumab	Clinical Trial	2015	PD-L1-positive, advanced NSCLC	No OS benefit from Pembrolizumab over Docetaxel in EGFR mutant vs wild-type patients (HR: EGFR mutant-0.88(0.45-1.70) vs EGFR wild-type 0.66(0.55-0.80)).
Checkmate-012 (NCT01454102) (106)	Nivolumab	Clinical Trial	2016	First-Line Treatment of Advanced NSCLC	Compared with patients with the wild-type EGFR, those with EGFR mutations had mPFS of 1.8 months vs 6.6 months.
Checkmate-057 (NCT01673867) (107)	Nivolumab	Clinical Trial	2015	Advances in first-line platinum-based therapy for stage IIIB/IV non-squamous NSCLC.	Patients with EGFR mutations did not derive any benefit from Nivolumab in terms of either PFS or OS (HR: 1.18 (0.69–2.00)).
OAK (NCT02008227) (108)	Atezolizumab	Clinical Trial	2016	Advances in platinum-based therapy for stage IIIB/IV NSCLC.	No OS benefit from Atezolizumab over Docetaxel in EGFR mutant vs wild type patients (HR: EGFR mutant-1.24(0.71-2.18) vs EGFR wild-type 0.69 (0.57-0.83)).
Chee Khoon Lee, et al. (109)	Nivolumab; Pembrolizumab; Atezolizumab.	meta-analysis	2016	Randomized trials comparing ICIs against chemotherapy were identified.	In the EGFR wild-type PD-1/PD-L1 significantly prolonged OS, (HR = 0.66,95%CI=0.58–0.76, *P* < 0.0001), but not the EGFR-mutant subgroup (HR = 1.05,95%CI=0.70–1.55, *P* < 0.81; treatment-mutation interaction *P* = 0.03).
Chee Khoon Lee, et al. (110)	Nivolumab; Pembrolizumab; Atezolizumab.	meta-analysis	2017	Randomized trials comparing ICIs against chemotherapy were identified.	They prolonged overall survival in the EGFR wild-type subgroup (HR,0.67;95%CI,0.60-0.75; P <0.001), but not in the EGFR mutant subgroup (HR, 1.11; 95%CI,0.80-1.53; *P* = 0.54; interaction, *P* = 0.005).
ATLANTIC NCT02087423 (113)	Durvalumab	Clinical Trial	2018	Eligible patients had advanced NSCLC with disease progression following at least two previous systemic regimens.	The proportion of patients with EGFR−/ALK− NSCLC achieving a response was higher than the proportion with EGFR+/ALK+ NSCLC achieving a response. The clinical activity of durvalumab in patients with EGFR+ NSCLC with 25% of tumor cells expressing PD-L1 was encouraging.
WJOG8515L jRCTs051180133 (111)	Nivolumab	Clinical Trial	2021	locally advanced, metastatic, or recurrent non-squamous NSCLC positive for an activating mutation of EGFR	The mPFS were 1.7 months for Nivolumab vs 5.6 months for Carboplatin-pemetrexed [log-rank *P* < 0.001; HR:1.92, with a 60%CI of 1.61–2.29].
Morimoto et al. (112)	Immune checkpoint inhibitor	retrospective	2022	Histologically confirmed NSCLC, confirmed EGFR-activating mutation	The mPFS and mOS were significantly longer with the chemo-immunotherapy regimen than with the ICI monotherapy regimen (5.7 months vs 1.5 months, *P* = 0.001, and 18.2 months vs 4.9 months, *P* = 0.001).

NSCLC, Non-Small Cell Lung Cancer; EGFR, epidermal growth factor receptor; ALK, Anaplastic Lymphoma Kinase; TKI, tyrosine kinase inhibitor; ICIs,immune checkpoint inhibitors; PD-L1, programmed death 1; PD-L1, programmed death-ligand 1; mPFS, median progression-free survival; mOS, median overall survival; HR, hazard ratio; CI, confidence interval.

The inherently low TMB and immunosuppressive microenvironment in patients with EGFR-mutated NSCLC are the primary reasons for the poor efficacy of monotherapy with ICIs (114), while PD-L1 expression, although it has some influence, is insufficient to reverse this disadvantage. Exploring more optimized combination therapy regimens is key to overcoming the current treatment bottleneck associated with EGFR-TKI resistance.

### 6.2 Immunotherapy combined with EGFR-TKIs

In first-line treatment, the combination of immunotherapy with EGFR-TKIs did not significantly improve efficacy and increased adverse events (AEs) (**Table 2**). In the KEYNOTE-021 trial, patients receiving Pembrolizumab in combination with Gefitinib discontinued treatment due to severe hepatotoxicity, and the incidence of Grade 3 or higher AEs reached as high as 71.5% (115). Clinical studies of ICIs combined with EGFR-TKI in later-line settings have yielded similar findings. In the CheckMate-012 study, mPFS was 5.1 months, and mOS was 18.7 months, with no significant increase in efficacy or AEs (116). However, the TATTON, CAURAL, and NCT02088112 clinical trials were all forced to terminate due to serious AEs, with severe interstitial pneumonia and liver injury being the primary AEs (117–119). In the NCT02013219 and CheckMate-370 studies, first-line combination therapy with immunotherapy and EGFR-TKIs in patients with ALK-positive NSCLC also demonstrated serious adverse reactions (120, 121).

**Table 2:** Efficacy of immunotherapy combined with EGFR-TKI in patients with EGFR mutations.

Study	Drugs	Study type	Published year	Efficacy outcomes	Grade ≥3 AEs rate (%)
Keynote-021 (NCT02039674) (115)	Pembrolizumab+ Erlotinib Pembrolizumab+ Gefitinib	Clinical Trial	2018	mPFS: 19.5 months; mOS: NR mPFS: 1.4 months; mOS: 13.0 months	33.3% 71.5%
CheckMate012 (NCT01454102) (163)	Nivolumab+ Erlotinib	Clinical Trial	2018	mPFS: 5.1 months; mOS: 18.7 months	24%
TATTON (NCT02143466) (118)	Durvalumab(3or10mg/kg) +Osimertinib	Clinical Trial	2022	ORR: 43%; mDOR:20.4 months	48%
	first-line Durvalumab(10mg/kg) +Osimertinib			ORR:82%; mDOR: 7.1 months mPFS:9.0 months	82%
CAURAL (NCT02454933) (117)	Durvalumab+Osimertinib	Clinical Trial	2019	ORR:64%, mPFS: NR	8%
	Osimertinib			ORR:80%, mPFS: 19.3 months	53%
NCT02088112 (119)	Durvalumab+Gefitinib	Clinical Trial	2020	ORR: 63.3%, mPFS:10.1 months	68.8%

EGFR, epidermal growth factor receptor; TKI, tyrosine kinase inhibitor; AEs, Adverse Events; mPFS, median progression-free survival; mOS, median overall survival; ORR,Overall Response Rate; mDOR, median Duration of Response; NR, not reached.

In current clinical trials combining immunotherapy with EGFR-TKIs, the combination therapy has not increased efficacy but has instead led to severe AEs (122). This may be related to the timing and sequence of administration. Therefore, it is crucial to further optimize the administration regimens and methods for combination immunotherapy and targeted therapy, while paying close attention to the severity and management of AEs. With the continuous emergence of new third-generation EGFR-TKIs and ongoing structural optimization, these agents are becoming more potent while reducing toxicity. Additionally, relevant studies have found that strong PD-L1 expression in patients with EGFR-mutated NSCLC is associated with a poor prognosis following EGFR-TKI treatment. Immunotherapy combined with targeted therapy holds promise as a novel treatment approach. Therefore, large-scale prospective studies are necessary to validate efficacy and evaluate toxicity.

### 6.3 Immunotherapy combined with chemotherapy

In first-line treatment, there is a significant difference in efficacy and EGFR-TKIs. In the CheckMate-012, IMpower130 study, the mPFS for the chemoimmunotherapy was only 4.8-7.0 months, and the mOS was 18.6-20.5months (123, 124); moreover, the incidence of AEs was higher compared to immunotherapy alone (106, 123, 124). Several clinical studies have investigated chemoimmunotherapy following resistance to EGFR-TKIs (**Table 3**). In the KEYNOTE-789 and CheckMate-722 studies, chemoimmunotherapy did not significantly prolong PFS or OS compared to chemotherapy alone; mPFS was only 5.6 months, and mOS ranged from 15.9 to 19.4 months (23, 125). In the KEYNOTE-789 study, in the PD-L1-positive subgroup, the Pembrolizumab plus chemotherapy group showed a trend toward improved OS (23). In the ORCHARD study, the efficacy of Durvalumab combined with chemotherapy was limited, with a mPFS of only 4.8 months (126). The CT-18 study suggests that for patients with EGFR-TKI-resistant EGFR mutations who are T790M-negative, the mPFS with Toripalimab plus chemotherapy was 7.0 months; in patients with high PD-L1 expression, PFS reached 8.3 months, and mOS was 23.5 months, indicating significant survival benefits. Furthermore, this study suggests that potential beneficiaries include patients with high PD-L1 expression, T790M-negative status, high TMB, TP53 mutations, and L858R mutations (23).

**Table 3:** Efficacy of ACP/ABCP in patients with EGFR mutations.

Study	Drugs	Study type	Published year	Efficacy outcomes	Grade ≥3 AEs rate (%)
KEYNOTE-789 (NCT03515837) (23)	ACP CP	Clinical Trial	2024	mPFS: 5.6 months; mOS: 15.9 months mPFS: 5.5 months; mOS: 14.7 months	43.7% 38.6%
CheckMate 722 (NCT02864251) (125)	ACP CP	Clinical Trial	2024	mPFS: 5.6 months; mOS: 19.4 months mPFS: 5.4 months; mOS: 15.9 months	44.7% 29.4%
ORCHARD (NCT03944772) (126)	ACP	Clinical Trial	2022	mPFS:4.8 months; mOS:23.4 months	36%
CT-18 (NCT03924050) (164)	ACP	Clinical Trial	2021	mPFS:7.0 months; mOS:23.5 months	65%
IMpower150 (NCT02366143) (128)	ABCP ACP BCP	Clinical Trial	2021	mOS:27.8 months mOS:14.9 months mOS:18.1 months	- - -
NEJ043 (jRCTs031190066) (129)	ABCP	Clinical Trial	2023	mPFS:7.4 months; mOS:23.1 months	93.3%
ORIENT-31 (NCT03802240) (132, 133)	ABCP ACP CP	Clinical Trial	2023	mPFS:7.2 months; mOS:21.1 months mPFS:5.5 months; mOS:20.5 months mPFS:4.3 months; mOS:19.2 months	56% 41% 49%
ATTLAS (NCT03991403) (134)	ABCP CP	Clinical Trial	2023	mPFS:8.48months; mOS:20.63 months mPFS:5.62months; mOS:20.27 months	40.4% 21.6%
GFPC 06–2018 (NCT04042558) (130, 131)	ABCP ACP	Clinical Trial	2024	mPFS:7.3 months; mOS:17.2 months mPFS:7.2 months; mOS:16.8 months	69.1% 51.4%

EGFR, epidermal growth factor receptor; TKI, tyrosine kinase inhibitor; AEs, Adverse Events; mPFS, median progression-free survival; mOS, median overall survival; CP, chemotherapy; BCP, chemotherapy combined with anti-angiogenic therapy; ACP, chemoimmunotherapy; ABCP, chemoimmunotherapy combined with anti-angiogenic therapy; ORR, overall response rate.

There is controversy regarding the efficacy and benefits of chemoimmunotherapy in patients who have developed resistance to EGFR-TKIs. Further exploration of this combination therapy is needed. Chemotherapy can enhance immunogenicity and improve the tumor-suppressive microenvironment; when combined with PD-(L)1 inhibitors, it can enhance the anti-tumor immune response (127). Following resistance to EGFR-TKIs, changes in the TME—including upregulated PD-L1 expression, increased TMB levels, and the L858R mutation—may help identify patient subgroups that are likely to benefit from immunotherapy, warranting further investigation.

### 6.4 Combination of chemotherapy and immunotherapy with anti-angiogenic therapy

In first-line treatment, the combination of chemoimmunotherapy with anti-angiogenic therapy has demonstrated some efficacy in patients with EGFR mutations. In the IMPOWER-150 study, among patients with EGFR-sensitive mutations, the mOS was 29.4 months in the chemoimmunotherapy with anti-angiogenic therapy (ABCP group) compared to 18.1 months in the chemotherapy plus anti-angiogenic therapy (BCP group) and 19.0 months in the chemoimmunotherapy (ACP group). Furthermore, in the EGFR-TKI-resistant population, mOS was 27.8 months in the ABCP group, 18.1 months in the BCP group, and 14.9 months in the ACP group (128). The NEJ043 and GFPC 06–2018 studies also suggest that a four-drug combination regimen may be effective in patients with EGFR-TKI resistance (129–131). This suggests that the four-drug combination regimen may demonstrate superior efficacy in EGFR-TKI-resistant patients. Although the ML-41701 study did not meet its primary endpoint, it demonstrated the feasibility of the ABCP four-drug combination regimen and suggested that PD-L1 expression may serve as a marker for identifying a subgroup with a favorable prognosis (129). In the ORIENT-31 study, mPFS was 7.2 months in the ABCP group, 5.5 months in the ACP group, and 4.3 months in the chemotherapy-alone (CP) group, and mOS was 21.1 months, 20.5 months, and 19.2 months, respectively. Compared with chemotherapy alone, the combination of Sintilimab with Bevacizumab and chemotherapy significantly extended PFS to 7.2 months, significantly reduced the risk of disease progression by 49%, and also demonstrated a trend toward OS benefit (132, 133). The subsequent ATTLAS study yielded similar findings, with the ABCP group demonstrating a higher ORR (69.5% vs. 41.9%), longer mPFS (8.48 months vs. 5.62 months), and longer mOS (20.63 months vs. 20.27 months) compared to the CP group (134). Furthermore, studies have shown that PD-L1 expression levels are associated with improved PFS, with patients exhibiting high PD-L1 expression deriving significant benefit (134). Several meta-analyses have shown that the four-drug regimen combined with ABCP results in improved OS (135, 136).

Studies have shown that the addition of anti-angiogenic agents can normalize tumor vasculature, leading to the activation of the immune system, promoting the differentiation of immune cells, and enhancing their function (137, 138). Therefore, the combination of ICIs and anti-angiogenic therapy can improve the TIME and enhance antitumor immune responses (137, 138). Although the ABCP four-drug combination therapy has demonstrated good efficacy in patients with EGFR-TKI resistance, this regimen should be used with caution, particularly in elderly patients, those in poor physical condition, or those with contraindications to Bevacizumab, following a thorough assessment of the patient’s general condition.

### 6.5 Other immunotherapy-related treatments

There are only small-sample-size studies on the combination of immunotherapy and anti-angiogenic therapy. A regimen combining Atezolizumab and Bevacizumab was explored, but the study was terminated early because only 3 patients achieved a partial response (PR) (139). The ALTER-L038 study evaluated the efficacy and safety of the anti-PD-L1 antibody Benmelstobart in combination with Anlotinib in patients with EGFR-positive advanced NSCLC who had progressed after EGFR-TKI treatment. It demonstrated an mPFS of 8.97 months, while mOS is not yet mature (currently 28.9 months); however, the ORR was only 25.5%, which was lower than expected (140). Further phase III trials are needed to identify the patient subgroups that may benefit most from this combination regimen.

Currently, two phase II clinical trials using PD-1-based bispecific antibodies have demonstrated some potential in patients who have developed resistance to EGFR-targeted therapy. The combination of Ivonescimab (a PD-1/VEGFR bispecific antibody) and chemotherapy achieved an ORR of 68.4% and a mPFS of 8.5 months in patients who had developed resistance to EGFR-TKIs (141). Iparomlimab and Tuvonralimab (a PD-1/CTLA-4 bispecific antibody) combined with anti-angiogenic therapy and chemotherapy achieved an ORR of 54.8% and a mPFS of 8.5 months in patients who had developed resistance to EGFR-TKIs, with good tolerability (142). There have been case reports of patients treated with Cadonilimab (a PD-1/CTLA-4 bispecific antibody) in later-line settings who showed progression on immunotherapy but may still have opportunities for re-treatment (143). Dual immunotherapy is a potential treatment option, but it currently requires validation through large-scale, multicenter clinical trials.

## 7 Future research directions and technical approaches

To address the bottlenecks in immunotherapy following the development of resistance to targeted therapies in advanced EGFR-mutated NSCLC, future research should focus on exploring innovative technical approaches to overcome current limitations in treatment efficacy.

### 7.1 Emerging combinations of antibody-drug conjugates and ICIs

Currently, ADCs such as T-DXd, Dato-DXd, Sac-TMT, and HER3-DXd offer new hope for overcoming resistance to third-generation TKIs and are expected to reshape the treatment landscape following resistance (144–147). Current research has shown that TROP-2-targeted ADCs can reprogram the TME through multiple signaling pathways, potentially enhancing antitumor immunity (148). Immunogenic cell death (ICD) induced by HER2-DXd and HER3-DXd can release tumor antigens and activate adaptive immunity (149, 150). ICIs work by blocking inhibitory signaling pathways such as PD-1/PD-L1 and CTLA-4, thereby reversing T-cell exhaustion and amplifying the anti-tumor immune response (151). ADCs transform “cold tumors” into “hot tumors”, thereby creating a more effective foundation for immune responses to ICIs (152). However, combination therapy requires close attention to the cumulative effects of AEs: both classes of drugs may cause immune-related adverse events (irAEs) or organ-specific toxicity (such as ADC-associated interstitial lung disease), and these adverse reactions require close monitoring and individualized management (153). Currently, safety data on combination use are limited, and there is an urgent need for further high-quality clinical studies to clarify the risk profile.

### 7.2 Combination of radiotherapy and ICIs

For patients with oligometastatic disease who have developed resistance to EGFR-TKIs, aggressive local treatment of progressive lesions combined with continued EGFR-TKI therapy can significantly improve PFS and OS (154, 155). Although there are few studies on the combination of radiotherapy and ICIs, it is well established that radiotherapy of oligometastatic lesions can enhance local control. SBRT can induce ICD, release damage-associated molecular patterns (DAMPs), activate dendritic cells, promote tumor antigen presentation, enhance CD8^+^ T-cell infiltration, and upregulate PD-L1 expression, thereby transforming local radiotherapy into a systemic antitumor immune response (156, 157). The regression of non-irradiated lesions following SBRT combined with ICI (i.e., the distant effect) suggests an “*in situ* vaccine” effect (157, 158). Furthermore, low-dose radiotherapy(LDRT)can reverse the “desert-like” TME and overcome immune resistance (159). LDRT promotes the infiltration and activation of immune cells by activating the interferon signaling pathway and upregulating various inflammation-related genes and chemokines (159). Concurrently, LDRT can reprogram myeloid cells in the TME, particularly DCs and macrophages, enabling them to present antigens more effectively and activate T cells (159). Despite these preclinical advantages, the optimal dose and fractionation regimen for radiotherapy, as well as the sequence of administration with ICIs, remain to be determined. The combination of radiotherapy and ICIs may synergistically increase the risk of irAEs, such as radiation pneumonitis, particularly in the irradiated lung regions; therefore, dose and treatment fields must be rigorously assessed. Overall, the combination of radiotherapy and ICIs warrants further investigation; however, critical gaps in clinical evidence must be addressed to establish its role in patients who have developed resistance to EGFR-TKIs.

### 7.3 Development of bispecific antibodies and novel immune checkpoints

By simultaneously targeting multiple immune regulatory pathways (such as PD-1/CTLA-4 or PD-1/TIM-3), bispecific antibodies hold promise for overcoming the immunosuppressive state of the TME (160). Research must elucidate the specific immune evasion mechanisms underlying EGFR mutations and identify new targets (such as LAG-3 and TIGIT) that can synergistically enhance T-cell activity (36). Additionally, the role of bispecific antibodies in reversing EGFR-mediated functional defects in dendritic cells (DCs) and impaired CD8+ T-cell activation must be validated (35, 47). However, such drugs have not yet been validated in clinical practice. Bispecific antibodies and novel ICIs also face practical challenges, including potential irAEs and uncertainty regarding the optimal dosage. Therefore, bispecific antibodies based on novel ICIs hold promise for the treatment of EGFR-TKI-resistant disease.

### 7.4 Combined epigenetic regulatory strategies

Epigenetic modifications (such as DNA methylation and histone modifications) play a critical role in shaping the immunosuppressive TME in EGFR-mutant NSCLC. Studies have shown that EGFR mutations can induce epigenetic reprogramming, thereby suppressing the expression of antigen-presentation-related genes (47, 89). Future research should explore the synergistic mechanisms between epigenetic modulators (e.g., DNMT inhibitors, HDAC inhibitors) and ICIs: restoring MHC class I molecule expression in tumor cells to enhance neoantigen presentation (160); reversing the infiltration and function of immunosuppressive cells (Tregs, MDSCs) (47); regulating the dynamic expression of PD-L1 to reverse immune checkpoint-mediated T-cell exhaustion (33, 89). In summary, the combined intervention of epigenetic regulation and immunotherapy, by reshaping the TIME and reversing mechanisms of resistance, shows promise in improving the prognosis of patients with EGFR-TKI resistance and represents a key area of current translational research.

### 7.5 Personalized vaccines and adoptive cell therapy

Personalized vaccines based on tumor-specific neoantigens represent a potential pathway to overcoming low immunogenicity. Research should capitalize on the unique genomic characteristics of EGFR-mutated NSCLC: using liquid biopsy to dynamically monitor the evolution of the neoantigen profile following drug resistance (84); designing peptide or mRNA vaccines targeting EGFR resistance mutations (e.g., C797S) (161); and exploring adoptive cell therapies using tumor-infiltrating lymphocytes (TILs) or genetically engineered T cells (e.g., CAR-T cells) to overcome T-cell functional suppression in the TME (47, 162). However, the clinical translation of personalized vaccines and Adoptive Cell Therapy still faces multiple challenges, including drug resistance heterogeneity, immunosuppression within the microenvironment, standardization of techniques, and the optimization of personalized treatment. In the future, it will be necessary to deepen mechanistic research, advance the clinical validation of combination strategies, and establish a precise biomarker system to guide personalized application. , , , , .

## 8 Discussion

ICIs generally demonstrate limited efficacy in patients with EGFR-mutated NSCLC, and the mechanisms underlying treatment failure can be analyzed from the following perspectives: 1. Abnormalities in the TIME: EGFR-mutant NSCLC tumors exhibit high heterogeneity in PD-L1 expression levels, TMB, and other immune microenvironmental characteristics, making them less conducive to ICI-mediated activation of antitumor immune responses compared to wild-type tumors (33). 2. Defects in the interferon (IFN) signaling pathway: IFN-γ downregulates the expression of MHC-I molecules, weakening the tumor’s antigen-presenting capacity and thereby inhibiting T-cell recognition and activation (165). 3. The impact of STK11 and KEAP1 mutations: Loss of STK11 function is associated with poor prognosis in NSCLC immunotherapy (166). KEAP1 mutations activate the NRF2 antioxidant pathway, leading to “immunologically cold” characteristics in the TME (such as reduced infiltration of dendritic cells and CD8^+^ T cells) and enhancing resistance to ferroptosis, thereby mediating ICI resistance (167). 4. Clonal Architecture and Co-mutation Complexity: Tumors with EGFR mutations are often accompanied by other genomic events (such as ARID1A, KEAP1, STK11, etc.), and their clonal evolution and co-mutation patterns may influence treatment response (168, 169).

Following resistance to EGFR-TKIs, changes occur in immune-related biomarkers and the TIME. Elevated expression of PD-L1, TMB, and CTLA-4, along with alterations in CD8+ T-cell infiltration and the number and activity of Tregs, further establish the theoretical basis for the use of ICIs in patients with EGFR-TKI resistance. However, treatment strategies can be optimized across different clinical scenarios: 1. For patients with no targeted treatment options following EGFR-TKI resistance, platinum-based chemotherapy combined with ICIs forms the foundational treatment framework. Multiple studies support that this regimen significantly improves objective response rates and survival benefits, particularly for patients with good performance status. 2. In specific subgroups (e.g., those with high tumor burden or rapid disease progression), adding anti-angiogenic agents (such as Bevacizumab) to the chemotherapy-immunotherapy combination regimen may enhance synergistic effects by modulating the TME; however, safety concerns, such as the risk of bleeding, must be comprehensively evaluated (131, 170). In addition, this regimen is particularly suitable for patients who have previously received TKI therapy and have no other standard treatment options (129). 3. For patients with brain metastases, although immunotherapy has demonstrated activity against brain lesions in some studies, its efficacy remains limited by the blood-brain barrier and the unique characteristics of the intracranial immune microenvironment. It should be combined with local treatments (radiation therapy/surgery) and closely monitored for neurological progression (171). 4. For treatment-resistant patients with high PD-L1 expression (TPS≥50%) and no risk factors for hyperprogression, monotherapy with an immunotherapy agent may be considered with caution; however, it should be noted that the response rate is significantly lower than that observed in patients with negative driver gene status, and patients should be monitored continuously for signs of hyperprogression (172). 5. For patients who cannot tolerate chemotherapy, a potential alternative is a chemotherapy-free combination regimen: PD-L1 inhibitors combined with multi-targeted anti-angiogenic agents (140).Following resistance to EGFR-TKIs, the TME changes, and immune markers increase; precision immunotherapy guided by molecular subtyping warrants further exploration: 1. Dynamic monitoring of PD-L1 expression: PD-L1 expression levels are an important but not the sole predictive marker. A re-biopsy is required to assess PD-L1 status after resistance, as targeted therapy may alter its expression. Patients with high PD-L1 expression are more likely to benefit from immunotherapy combination regimens, but for PD-L1-negative patients, other biomarkers still need to be explored. 2. TMB has limitations; NSCLC with EGFR mutations generally exhibits low TMB, which weakens its predictive value. A comprehensive evaluation should be conducted in conjunction with neoantigen profiling and immune gene expression profiles (e.g., IFN-γ signaling) (173). 3. Mechanisms of drug resistance are linked to the immune microenvironment; patients with secondary resistance mutations (such as T790M/C797S) may exhibit different immunogenicity profiles compared to those with bypass activation (such as MET amplification). Dynamic monitoring of the resistance mutation profile via liquid biopsy can provide a basis for determining the optimal timing of immunotherapy (161). 4. A specific co-mutation subtype: The EGFR/CDKN2A co-mutation subtype exhibits unique immune sensitivity, suggesting that molecular subtyping can further refine the identification of patients likely to benefit from immunotherapy (173).In later-line treatment, re-challenge with immunotherapy may be considered to consolidate the therapeutic benefits of ICIs. This is particularly applicable in the following situations: 1. Slow or minimal disease progression, with no rapid worsening of symptoms;2. Previous clinical benefit from ICI treatment; 3. No history of severe immune-related AEs; 4. Risk of rapid disease progression.

The purpose of this review is to explore the immune mechanisms underlying immune resistance and EGFR-TKI resistance in patients with EGFR mutations. It synthesizes existing immunotherapy regimens for patients with EGFR-TKI resistance, identifies potential beneficiary populations, and examines the advantages and disadvantages of different treatment modalities. We aim to provide a basis for precise clinical decision-making, standardize the clinical application of immunotherapy following EGFR-TKI resistance, assist in identifying high-benefit patient populations and optimizing treatment strategies, and guide future clinical research and technological advancements, ultimately improving the survival outcomes of patients with EGFR-TKI resistance.
